# Simple and Reliable Determination of the Histamine Content of Selected Greek Vegetables and Related Products in the Frame of “Low Histamine Diet”

**DOI:** 10.3390/foods11203234

**Published:** 2022-10-17

**Authors:** Apostolia Tsiasioti, Paraskevas D. Tzanavaras

**Affiliations:** Laboratory of Analytical Chemistry, School of Chemistry, Faculty of Sciences, Aristotle University of Thessaloniki, GR-54124 Thessaloniki, Greece

**Keywords:** histamine, low histamine diet, vegetables, food samples, post column derivatization

## Abstract

The determination of histamine in Greek foods that should potentially be avoided during a “low histamine diet” is reported herein. Cation exchange chromatography combined to selective post column derivatization proved to be an excellent tool for this type of analysis as well, offering accurate results following minimal sample preparation. Tomato-, eggplant- and spinach-related products have been successfully analyzed and were all found to contain histamine. Higher amounts were quantified in eggplants, eggplant salads and spinach in the range of 15.4–34.2 mg kg^−1^ and lower in fresh tomatoes and related products (0.8–10.6 mg kg^−1^). The method is capable of determining as low as 0.5 mg kg^−1^ histamine without matrix effects, with percent recoveries ranging between 87 and 112% (tomatoes and related products), 95 and 119% (eggplants and related products) and 90 and 106% (fresh and frozen spinach).

## 1. Introduction

A significant increase in the frequency of food intolerances tends to be a concerning phenomenon in most modern societies. Such disabling disorders provoke a critical decrease in the quality of life of this population [1,2,3]. Histamine intolerance (or food histaminosis or hypersensitivity to food histamine) is one characteristic example, arising from the failure of the diamine oxidase (DAO) enzyme to degrade dietary histamine at the intestinal level [4].

According to the European Food Safety Authority (EFSA) and Food and Agriculture Organization of the United Nations (FAO)/World Health Organization (WHO), histamine intolerance is associated with increased plasma histamine levels and is recognized as clinically different from the more established histamine intoxication [5]. Intoxication typically appears after the consumption of foods high in histamine, while intolerance is due to a deficiency in histamine metabolism, where symptoms can be triggered even by the consumption of low amounts [6].

A recent trend in the treatment of histamine intolerance is the concept of “low histamine diets” [5]. These diets usually exclude foods that have been proven to initiate intolerance symptoms to patients [7]. Such foods tend to be rich in histamine, but some, surprisingly, are not usually regarded as sources of this amine. Most high histamine foods are also considered to be high in protein (cheese, cured meats, etc.) [8], but there are also various widely consumed low protein plant foods that are reported to contain naturally higher amounts of the precursor amino acid histidine (spinach, tomatoes, and eggplants are representative examples, being more susceptible to histamine accumulation than other plant foods [9]); bacterial decarboxylation of the latter is considered to be the main source of histamine [10,11,12].

In previous reports from our group we have demonstrated that cation exchange chromatography coupled to a specific post column derivatization reaction offers unique advantages in terms of sensitivity and selectivity for the determination of histamine (and its precursor histidine when needed) in various complex matrices (biological material, fisheries, etc.) [7,13]. Compared to “traditional” reversed phase C18 columns, modern cation exchange analytical columns offer superior robustness, stability and endurance when complicated matrixes are injected without exhaustive purification [14]. Additionally, the unique post column reaction of the analytes with o-phthalaldehyde in the absence of nucleophilic reagents offers matrix effect-free analysis [15].

In the current communication we aim to expand the applicability of our aforementioned analytical protocols to the determination of plant-based fresh and processed widely-consumed Greek commercial foods. Our goal is to reliably determine and report their histamine content in the frame of the concept of “low histamine diet”. The high pressure liquid chromatography coupled to post column derivatization (HPLC-PCD) approach has been revalidated to examine potential matrix effects from the new substrates and the preparation of the samples has also been optimized to ensure the highest extraction yield of the analyte.

## 2. Materials and Methods

### 2.1. Instrumentation

The HPLC instrumentation consisted of an AS3000 autosampler (Thermo Scientific, Walltham, MA, USA); a LC-9A binary pump (Shimadzu, Kyoto, Japan); a RF-551 spectrofluorimetric detector operated at high sensitivity (Shimadzu, Kyoto, Japan); and an Elite^TM^ vacuum degasser (Alltech, Athens, Greece). A Minipuls^TM^ 3 peristaltic pump (Gilson, Middleton, WI, USA) was used for delivering the reagents. Two column ovens (Jones Chromatography and HiChrom Limited) were utilized in order to maintain the HPLC column and the reaction coil at the required temperatures. Chromatographic separations were performed by using a MetroSep C4 column (150 × 4.0 mm i.d., 5 μm) (Metrohm, Herisau, Switzerland). The PCD reaction coil (200 cm, tightly knitted around a stainless-steel rod) and connections were made of PTFE tubing (i.d. = 0.5 mm). Data acquisition was carried out using the Clarity^®^ software (version 4.0.3, DataApex, Prague, Czech Republic). Ultrasonic-assisted extraction of the analytes was carried out using an Elma Transsonic 460/H bath (Singen, Germany).

### 2.2. Reagents and Solutions

All reagents used throughout this study were of analytical grade and have been purchased from either Merck or Fluka. Doubly de-ionized water was produced by a Milli-Q system (Millipore, Thessaloniki, Greece).

1000 mg L^−1^ solutions histamine were prepared in HNO_3_ (7 mmol L^−1^), while working standards were diluted daily in the same solvent. O-phthalaldehyde (OPA) was prepared at 10 mmol L^−1^ by dissolution in 5 mL methanol following by dilution to 100 mL with doubly de-ionized water and was typically consumed within 1–2 working days (the reagent can be stable for at least 3–4 working days if kept refrigerated) [16]. Phosphate buffer (100 mmol L^−1^, pH = 9) was adjusted by drop-wise addition of 2.0 mol L^−1^ NaOH solution. The mobile phase (7 mmol L^−1^ HNO_3_) was prepared daily, including ultrasonic degassing and filtration under vacuum through 0.45 μm membrane filters (Whatman^®^, Athens, Greece).

### 2.3. HPLC-PCD Procedure

Twenty microliters of samples or standards were injected in the cation exchange column (thermostated at 60 °C) and were separated with isocratic elution (7 mmol L^−1^ nitric acid) at a flow rate of 1.0 mL min^−1^. Following elution, the analytes were mixed online with the streams (0.25 mL min^−1^ each stream) containing the derivatization reagents (OPA, 10 mmol L^−1^ and phosphate buffer, 100 mmol L^−1^/pH = 9). The OPA-derivatives were formed on passage through a tightly knitted thermostated reaction coil (200 cm/60 °C) and were delivered downstream to the fluorescence detector (360/440 nm). Peak area was employed for quantification in all cases.

### 2.4. Preparation of Samples

Commercially available fresh tomatoes, eggplants and spinach were purchased from local marketplaces. Related processed products such as tomato ketchup, tomato pastes, concentrated tomato juice, pre-cooked tomato spaghetti sauce and eggplant salads were also purchased from local supermarkets. All samples were stored as for everyday use until analysis (typically refrigerated at 4–7 °C) [17]. 

Sample processing/preparation was based on ultrasonic-assisted extraction in HNO_3_. In brief, accurately weighed amounts of each sample (ca. 20 g) were thoroughly mixed with 100 mL of HNO_3_ (50 mmol L^−1^) using a laboratory blender. The resulting mixture was extracted following ultrasonication for 15 min. Subsequent simple steps included centrifugation, filtration through syringe filters and HPLC-PCD analysis (either directly or after dilution with mobile phase when necessary). 

Similar preparation of pooled samples was adopted during the validation of the matrix effect of the method; representative pooled matrices included (i) tomato-based (fresh tomatoes, ketchup, tomato paste, tomato sauce), eggplant-based (various fresh eggplants and eggplant salads) and spinach-based (various fresh and frozen spinaches). 

Portions of selected representative samples (ketchup, tomato spaghetti sauce, tomato paste and eggplant salad) were also stored at room temperature in airtight food-compatible containers for stability studies.

## 3. Results and Discussion

The proposal, by our group, of cation exchange chromatography combined with online post column derivatization with OPA in the absence of nucleophillic compounds [18,19] has proven to be a reliable and robust analytical tool for the determination of histamine in complex matrices [7,13]. The specificity of the PCD reaction and the robustness of IEC require minimal sample processing, while sensitive fluorescence detection offers adequate analytical figures of merit that enable the direct quantification of endogenous histamine levels at the sub-mg kg^−1^ level.

### 3.1. Sample Preparation

Although there were no complicated extraction or purification steps involved in this work, it was necessary to investigate the optimal conditions that ensure efficient transfer of the polar analyte from complex food matrices [20]. Three pooled samples were prepared as for (i) tomato-based (fresh tomatoes, ketchup, tomato paste, tomato spaghetti sauce), (ii) eggplant-based (fresh eggplants and eggplant-based salads) and (iii) fresh and frozen spinach. 

**Figure 1:**
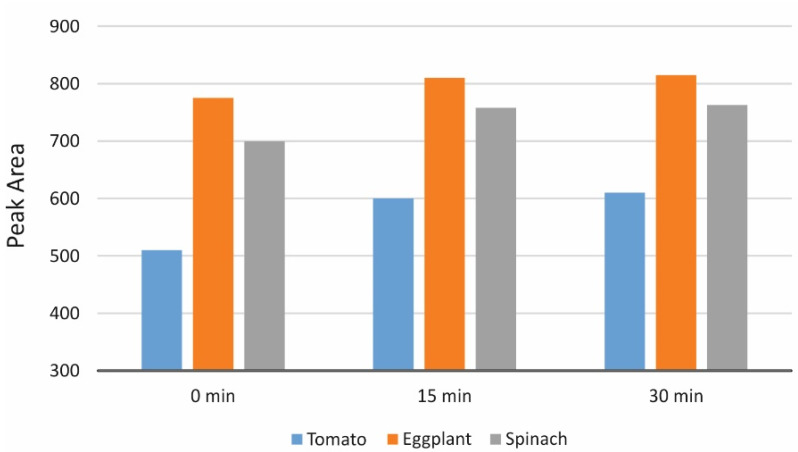
Effect of ultrasonication time on the extraction of histamine from three representative pooled matrices (tomato-, eggplant- and spinach-based). For experimental conditions please see Section 2.4 and Section 3.2.

Twenty grams of each pooled sample were dispersed in 100 mL of HNO_3_ (50 mmol L^−1^) and were blended for 3 min using a laboratory blender. The resulting mixtures were analyzed either directly or following ultrasonication for 15 and 30 min. As can be seen in the experimental results of Figure 1, 15 min of ultrasonic-assisted extraction were adequate for maximum histamine release from the matrix. 

Since the final step prior to HPLC injection involves filtration through PTFE syringe filters (polytetrafluoroethylene), it was necessary to confirm the absence of binding of the analyte on the membrane material [21]. A series of experiments using low-medium-high concentrations of aqueous standards of histamine confirmed the suitability of the selected filters for the extraction of the polar analytes with percent recoveries being in the range of 97–101%.

### 3.2. Evaluation of the Matrix Effect

In order to analyze efficiently complicated samples with minimal pretreatment, it is necessary to evaluate potential matrix effects versus the aqueous calibration curves [22]. Three pooled matrices were prepared independently for this purpose as described in Section 3.1 (tomato-, eggplant- and spinach-based respectively) and treated according to the procedure followed in Section 2.4. The resulting solutions were spiked with elevated amounts of histamine and the slopes of the respective matrix-matched calibration curves were compared versus the aqueous one. 

The experimental results presented in Table 1 confirmed the absence of matrix effects despite the simple sample processing, with the experimental values being in the range of −5.2 (tomato-based pooled matrix) to +4.9% (spinach-based pooled matrix).

### 3.3. Analytical Figures of Merit

Revalidation experiments confirmed linearity for the determination of histamine in the range of 0.5 to 10 mg kg^−1^ (*r* > 0.998), with an LOD of 0.1 mg kg^−1^. The percent residuals for six calibration points were better than ±12%.

Within-day precision ranged between 1.2 and 3.8%, while between-days precision was better than 6% (calculated as the RSD of the slopes of 6 consecutive calibration curves).

The accuracy was evaluated following spiking of the three pooled matrices used for the matrix effect evaluation with elevated amounts of histamine. The aqueous calibration curve was used for quantification. As shown in Table 2, the percent recoveries ranged between 87 and 112% for the tomato-based matrix, 95 and 119% for the eggplant-based matrix and 90–106% for the spinach-based matrix, respectively.

### 3.4. Analysis of Real Samples

**Figure 2:**
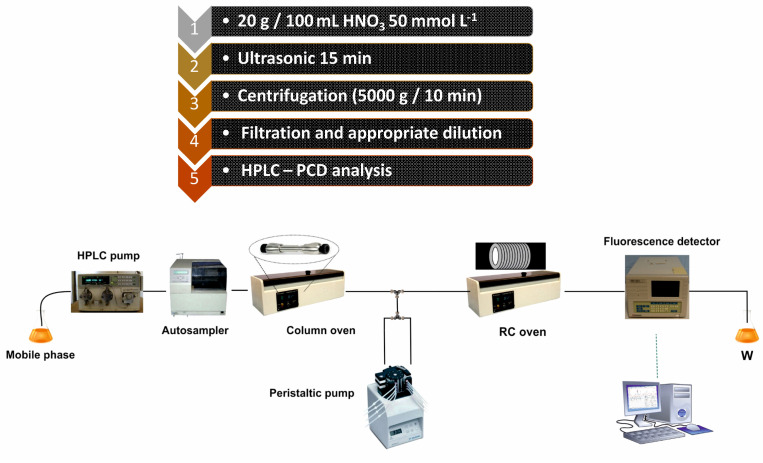
Typical workflow of the proposed analytical scheme.

**Figure 3:**
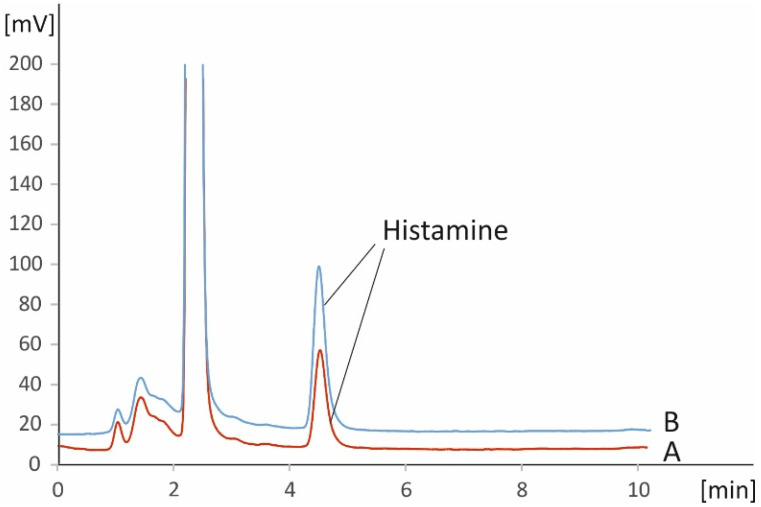
Representative chromatograms of an un-spiked (A) and spiked (B) real ketchup sample using the proposed IC-PCD method.

**Figure 4:**
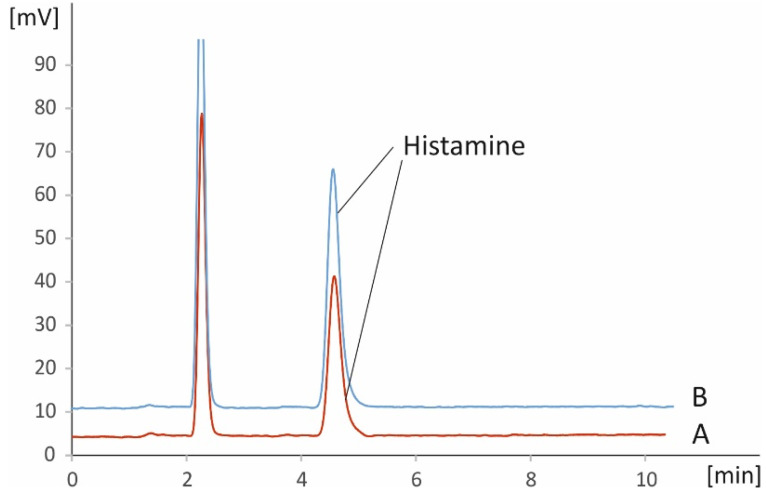
Representative chromatograms of an un-spiked (A) and spiked (B) real eggplant salad sample using the proposed IC-PCD method.

A graphical depiction of the workflow (sample preparation and analysis) of the method is shown in Figure 2. The results from the analysis of real samples can be found in Table 3 and Table 4. Table 3 includes data from fresh and frozen vegetables, while in Table 4 results from analysis of related products are presented. While endogenous histamine was quantified in all samples, fresh tomatoes were found to comparatively contain the lowest amounts of the analyte at levels around 1 mg kg^−1^, while significantly higher levels were found in eggplants (ca. 15–35 mg kg^−1^) and spinach (ca. 18–34 mg kg^−1^). Tomato-based processed products and especially pastes were found to be richer in histamine compared to fresh tomatoes, with histamine levels being higher than 2.5 mg kg^−1^. All quantitative results are in good agreement with previous reports [1,4,23]. Representative chromatograms from the presence of endogenous histamine in selected representative matrices (commercially available ketchup and eggplant salads) are depicted in Figure 3 and Figure 4. The respective figures also include chromatograms after spiking the matrices with known amounts of histamine. 

The effect of storage time on the levels of histamine in representative vegetable-related products (see Section 2.4) was evaluated upon storage at room temperature for five days in airtight containers suitable for food storage. None of the selected products showed significant alterations on the levels of histamine, with percent variations ranging between −13 and +5%, as can be seen in Table 5.

## 4. Conclusions

Cation exchange chromatography coupled to online post column derivatization of histamine with OPA in the absence of nucleophilic compounds has been demonstrated to be a reliable and advantageous analytical tool for the quantification of the analyte in various vegetables and vegetable-based processed food products. The absence of matrix effects or interferences in combinations with minimal sample processing establish the method as ideal for the large-scale screening of food samples in the frame of “low histamine diet”. Ongoing research intends to launch a database offering related data from widely consumed products in the Greek market to allergists.

## Figures and Tables

**Table 1:** Evaluation of the matrix effect (ME%).

	Aqueous	Tomato-Based Matrix	Eggplant-Based Matrix	Spinach-Based Matrix
**Slope**	0.480 (±0.008)	0.455 (±0.007)	0.498 (±0.010)	0.503 (±0.010)
**ME %**		−5.2%	+3.7%	+4.9%

**Table 2:** Accuracy of the proposed method for the analysis of histamine in real samples.

Samples	Spiked (μmol L^−1^)	% Recovery
**Tomato-based matrix**	1.0	112
	2.5	87
	5.0	92
	10.0	95
**Eggplant-based matrix**	1.0	119
	2.5	101
	5.0	95
	10.0	105
**Spinach-based matrix**	1.0	90
	2.5	106
	5.0	104
	10.0	105

**Table 3:** Histamine content in selected fresh/frozen vegetables.

Fresh Tomatoes	Histamine [mg kg^−1^] (±S.D.)
Sample 1	1.4 (±0.1)
Sample 2	1.0 (±0.9)
Sample 3	0.90 (±0.11)
Sample 4	0.93 (±0.09)
Sample 5	0.82 (±0.06)
Fresh Eggplants *	**Histamine [mg kg^−1^] (±S.D.)**
Sample 1	34.2 (±3.1)
Sample 2	23.2 (±1.5)
Sample 3	15.4 (±0.9)
Sample 4	21.4 (±1.2)
Sample 5	25.2 (±1.7)
Spinach *	**Histamine [mg kg^−1^] (±S.D.)**
Sample 1 (fresh)	30.2 (±2.9)
Sample 2 (frozen)	33.7 (±2.1)
Sample 3 (frozen)	17.9 (±1.0)

* Eggplant and spinach samples were diluted 5-fold prior to analysis.

**Table 4:** Histamine content in tomato- and eggplant-related products.

Tomato-Based Products	Histamine [mg kg^−1^] (±S.D.)
Tomato ketchup 1	1.1 (±0.07)
Tomato ketchup 2	1.0 (±0.06)
Slightly concentrated tomato juice 1	0.94 (±0.11)
Slightly concentrated tomato juice 2	0.81 (±0.05)
Salsa Napoletana	1.8 (±0.12)
Tomato paste 1	2.7 (±0.15)
Tomato paste 2	4.8 (±0.16)
Tomato paste double concentrated 1	10.6 (±0.35)
Tomato paste double concentrated 2	6.6 (±0.28)
Eggplant-Based Products *	**Histamine [mg kg^−1^] (±S.D.)**
Eggplant salad 1	23.0 (±0.9)
Eggplant salad 2	27.6 (±0.7)

* Eggplant salads samples were diluted 5-fold prior to analysis.

**Table 5:** Histamine content in tomato- and eggplant-related products after storage for 5 days at room temperature.

Stability Samples	% Variations of Histamine
Tomato ketchup	+5
Salsa Napoletana	−1.3
Tomato paste	+3
Eggplant salad	−3

## Data Availability

Data available on request.
